# Evolutionary analysis of CD300A and CD300C paired receptors in primates

**DOI:** 10.3389/fimmu.2025.1633323

**Published:** 2025-09-05

**Authors:** Carolina Dias, Francesca Levi-Schaffer, Pedro José Esteves

**Affiliations:** ^1^ Centro de Investigação em Biodiversidade e Recursos Genéticos (CIBIO), Research Network in Biodiversity and Evolutionary Biology (InBIO), Research Network in Biodiversity and Evolutionary Biology, Universidade do Porto, Porto, Portugal; ^2^ Departamento de Biologia, Faculdade de Ciências da Universidade do Porto, Porto, Portugal; ^3^ Pharmacology and Experimental Therapeutics Unit, Institute for Drug Research, School of Pharmacy, Faculty of Medicine, The Hebrew University of Jerusalem, Jerusalem, Israel; ^4^ CITS-Center of Investigation in Health Technologies, Cooperativa de Ensino Poltécnico e Universitário (CESPU), Gandra, Portugal

**Keywords:** CD300, CD300a, CD300c, primates, evolution, multigene family

## Abstract

**Introdction:**

The CD300 family comprises immunoglobulin superfamily receptors that regulate immune cell function through inhibitory or activating signals. CD300A and CD300C form a paired receptor system that recognizes shared ligands but mediates opposing effects: CD300A transduces inhibitory signals, whereas CD300C promotes activation.

**Methods:**

Here, we investigated the evolutionary history of these receptors in primates by analyzing 62 sequences from 33 primate species.

**Results:**

Using phylogenetic reconstruction, synteny analysis, functional motif conservation, and pseudogene identification, we found that CD300A is present in all species examined. In contrast, CD300C exhibits a dynamic evolutionary profile, with multiple independent pseudogenization events, functional impairments, and complete loss in some lineages like Hylobatidae and some species like *Lemur catta*.

**Discussion:**

These contrasting patterns suggest that while CD300A plays an essential and irreplaceable role in immune regulation, the activating function of CD300C may be context-dependent or dispensable. Additionally, we identified evidence of gene conversion between CD300A and CD300C in several lineages, preserving extracellular domain similarity despite divergent signaling functions. Our findings highlight the complex evolutionary dynamics of the CD300 gene family and provide new insights into how primate immune systems adapt.

## Introduction

Leukocyte membrane molecules allow immune cells to sense their surrounding environment, triggering responses that either contribute to the maintenance of homeostasis or initiate an inflammatory response in the presence of infection or tissue damage. To regulate the immune response, a balance between inhibitory and activating receptors allows the immune system to combat invading organisms while minimizing host damage (1–3).

The CD300 molecules are a family of immunoreceptors that belong to the immunoglobulin superfamily. In humans, the CD300 immunoreceptor family consists of eight members (CD300A, LB, C, LD, E, LF, LG, and H), located on chromosome 17q25.1 (1, 4, 5). The CD300 molecules are type I transmembrane glycoproteins, characterized by a single extracellular IgV-like domain, a transmembrane region, and a cytoplasmic tail (1–4, 6). The CD300A and CD300C receptors are paired receptors, a class of structurally similar molecules that recognize the same ligands but exert opposing functions, with CD300A functioning as an inhibitory receptor and CD300C as an activating receptor. Having a similar extracellular domain, they bind to the same ligands, while differences in their intracellular signaling lead to distinct immune responses (7, 8).

The human CD300A molecule consists of a N-terminal signal peptide sequence (1-17), an extracellular domain (18-180), a transmembrane domain (181-201), and a cytoplasmic tail (202-299). Within the extracellular region, a disulfide bond (C36↔C103) and two N-glycosylation sites at N83 and N92 are present. The segment of amino acids linking the ectodomain to the transmembrane region is abundant in proline, serine, and threonine residues (2).The long cytoplasmic tail of CD300A contains four ITIMS (immunoreceptor tyrosine-based inhibitory motifs). Three of these conform to the consensus sequence for classic ITIMs (I/V/L/SxYxxL/V), while the fourth follows a non-classical motif (I/V/L/S/TxYxxL/V/I). Upon activation, these motifs recruit phosphatases, such as SHP-1 and SHP-2, leading to inhibitory signaling and modulation of leukocyte function (9).

**Figure 1:**
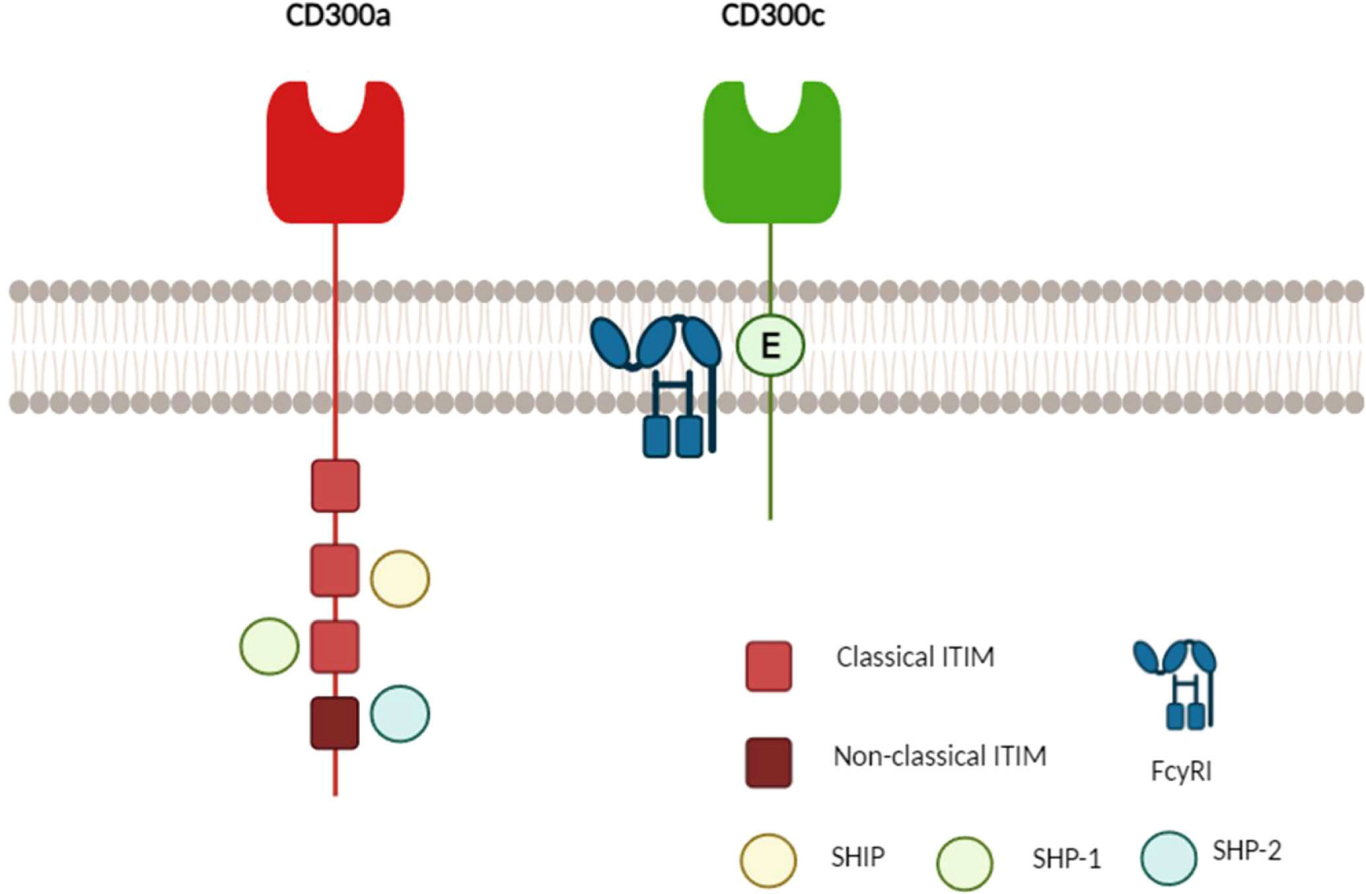
Schematic representation of the molecular structure of human CD300A and CD300C. CD300A (red) possesses a long cytoplasmic tail containing four immunoreceptor tyrosine-based inhibitory motifs (ITIMs): three classical (bright red) and one non-classical (dark red). Upon phosphorylation, these ITIMs recruit phosphatases such as SHIP, SHP-1, and SHP-2, leading to inhibitory signaling. In contrast, CD300C (green) has a short cytoplasmic tail that lacks immunoreceptor tyrosine-based activation motifs (ITAMs) and instead interacts with adaptor proteins such as FcγRI through a charged glutamic acid residue (E), facilitating activating signaling (1, 9, 13, 14).

The human CD300C molecule features an N-terminal signal peptide sequence (1-20), an extracellular domain (21-183), a transmembrane domain (184-204), and a cytoplasmic domain (205-224). The extracellular region contains two disulfide bonds (C43↔C110 and C57↔C65), along with N-glycosylation sites at N90 and N99 and O-glycosylation sites at T136, T137, and T149. Unlike CD300A, the CD300C cytoplasmic tail is short and lacks intrinsic signaling motifs. Instead, its transmembrane domain contains a charged glutamic acid residue, which facilitates interactions with adaptor proteins containing ITAMs (immunoreceptor tyrosine-based activating motifs), such as FcγRI, leading to the activation of immune signaling pathways (**Figure 1**) (1, 10–12).

CD300A and CD300C recognize phospholipids exposed on apoptotic or activated cells. Both receptors interact with phosphatidylserine (PS) and phosphatidylethanolamine (PE), with CD300A displaying a higher binding affinity for PE compared to CD300C (6). This difference in PE recognition is attributed to specific amino acid residues: F56-L57 in CD300A and L63-R64 in CD300C (12). While CD300C binds to PS and PE at similar levels, CD300A exhibits a stronger interaction with PE while maintaining comparable interactions with PS. Additionally, the ligand-binding affinity of CD300C is weaker than that of CD300A, a pattern commonly observed in paired receptors, where inhibitory receptors tend to establish stronger ligand interactions (14).

The birth-and-death model of evolution has been proposed as a mechanism for the evolution of multigene families, where new genes arise through gene duplication events. Among the duplicated genes, some are retained in the genome and undergo functional divergence via neofunctionalization, while others accumulate deleterious mutations and become pseudogenes or are eventually deleted from the genome. This evolutionary process leads to multigene families that consist of both divergent gene groups and highly homologous genes within those groups, as well as pseudogenes. This model is particularly relevant to immune system gene families, such as immunoglobulins, where it explains the generation of functional diversity essential for immune defense (15, 16).

Primates originated from a common ancestor approximately 85 million years ago during the Late Cretaceous/Early Paleocene. During the Eocene period, the diversification of primates resulted in the emergence of three main lineages: 1) Strepsirrhini, which diverged around 68.7 MYA and includes Lorisiformes (galagos, pottos, lorises) which originated around 40.3 MYA, Chiromyiformes (Malagasy aye-aye) with an origin around 58.6 MYA, and Lemuriformes (Malagasy lemurs) with an origin around 38.6 MYA; 2) Tarsiiformes which split from other primates approximately 81.3 MYA and are now represented by tarsiers; and Simiiformes, which emerged around 43.5 MYA, and later diverged into Platyrrhini (New World monkeys) and Catarrhini (Old World monkeys and hominoids), with their separation from a common ancestor occurring approximately 24.8 MYA. Within Catarrhini, further diversification gave rise to Cercopithecoidea (Old World monkeys) around 18 MYA and Hominoidea (humans, great apes, and gibbons) around 20 MYA (17).

Despite their critical role in immune regulation, the evolutionary history of CD300A and CD300C remains largely unexplored, even in primates. Understanding the evolutionary trajectories of paired receptors like CD300A and CD300C is essential for uncovering the mechanisms that drive immune receptor diversification and adaptation, shedding light on how primates have evolved to balance immune activation and inhibition in response to diverse pathogenic pressures.

## Methods

### Data retrieval and sequence selection

A total of 62 CD300A and CD300C sequences, also referred to as CMRF35-like molecules 8 and 6 respectively, from 33 primate species were retrieved from the National Center for Biotechnology Information (NCBI) GenBank database. Sequence retrieval was initiated with BLAST searches using the human CD300A (NM_007261) and CD300C (NM_006678) reference sequences as queries. Subsequently, additional BLAST searches were performed using representative sequences from major primate groups to identify orthologs that may not have appeared in the initial query. Additionally, CD300 sequences from *Tupaia chinensis* were included as outgroups to provide a broader evolutionary perspective.

### Synteny analysis

Synteny analysis was performed using the NCBI Genome Data Viewer to investigate the genomic organization of the CD300 genes. Orthologs were further validated by conducting BLAST searches in public databases (NCBI BLASTn) comparing the sequences across species to confirm homology. Genes adjacent to the CD300 gene cluster (*GPRC5C* and *RAB37*) were used as genomic anchors to assess the conservation of synteny.

### Sequence alignment and phylogenetic analysis

Sequence alignments were performed using the ClustalW Multiple Alignment tool implemented in the BioEdit Sequence Alignment Editor. Manual adjustments were applied to maintain correct reading frames and improve alignment quality, particularly in regions with potential frameshifts or indels.

Phylogenetic analyses were performed in MEGA X (version 11.0.13) using the maximum likelihood (ML) method. The amino acid phylogenetic tree was constructed based on the highly homologous portion of the extracellular domain (around 112 amino acids) of CD300A and CD300C, representing the most conserved region among CD300 genes. The optimal nucleotide substitution model for the dataset was determined in MEGA X to be JTT+G with five gamma-distributed rate categories. Bootstrap analysis with 1000 replicates was conducted to evaluate the trees’ robustness.

### Amino acid variability, ITIMs conservation, and pseudogenization analysis

To assess amino acid variability, separate multiple sequence alignments were conducted for each gene using their complete coding sequences. These alignments allowed the identification and analysis of conserved cysteine residues (involved in disulfide bond formation), glycosylation sites, and potential pseudogenization events (premature stop codons or frameshift mutations). Sequence variability was analyzed across primate families to identify evolutionary patterns potentially linked to ecological adaptations or selective pressures.

**Figure 2:**
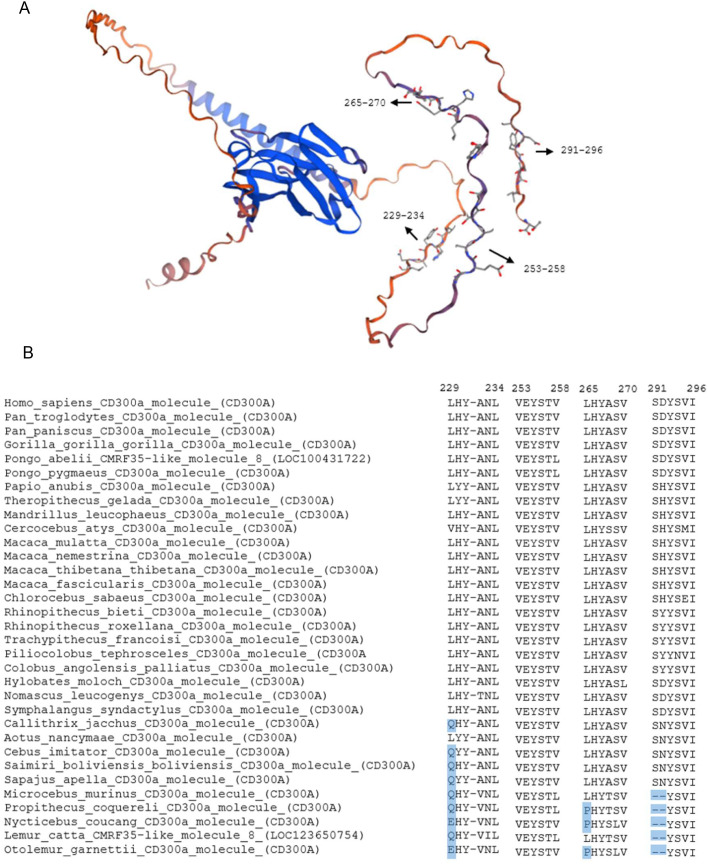
Structure model of human CD300a and conservation of CD300a ITIMs across primates. **(A)** Structural model of the human CD300a. Black arrows indicate the position of immunoreceptor tyrosine-based inhibitory motifs (ITIMs), with the corresponding amino acid positions labeled according to the human sequence. **(B)** Multiple sequence alignment of CD300A ITIM regions across various primate species, with the numbers indicating the corresponding amino acid position in the human sequence. Dashes (-) indicate gaps in the sequence alignment.

The conservation of ITIM sequences in CD300A and the charged glutamic acid residue in CD300C was also evaluated. These analyses aimed to determine the degree of conservation of the ITIM consensus sequence and the critical transmembrane residue in CD300C, identifying sequence variations that could alter, disrupt, or abolish receptor signaling and ultimately affect their immune regulatory function. The human CD300A and CD300C three-dimensional models (**Figure 2A**) were obtained with Swiss model using the sequences from Uniprot (accession numbers: Q9UGN4 and Q08708 respectively).

## Results/discussion

An evolutionary analysis of CD300A and CD300C was conducted across 33 primate species, retrieving a total of 63 gene sequences from the NCBI GenBank database. Phylogenetic analyses (**Supplementary Figure 1**) were performed for each gene. To assess potential pseudogenization events, premature stop codons or frameshift mutations were manually annotated based on alignment results and used as indicators of gene inactivation. The gene distribution across species is summarized in **Table 1**.

**Table 1:** Distribution of CD300A and CD300C in primate species.

Family	Species	CD300A	CD300C
Cercopithecidae	Papio anubis	1	1
	Theropithecus gelada	1	1
	Mandrillus leucophaeus	1	1
	Cercocebus atys	1	1
	Macaca mulatta	1	1
	Macaca thibetana thibetana	1	1
	Macaca fascicularis	1	1
	Macaca nemestrina	1	1
	Chlorocebus sabaeus	1	1
	Rhinopithecus bieti	1	1*
	Rhinopithecus roxellana	1	1
	Trachypithecus francoisi	1	1
	Piliocolobus tephrosceles	1	1
	Colobus angolensis palliatus	1	1
Hominidae	Pan paniscus	1	1
	Pan troglodytes	1	1*
	Homo sapiens	1	1
	Gorilla gorilla gorila	1	1*
	Pongo abelii	1	1
	Pongo pygmaeus	1	1
Hylobatidae	Hylobates Moloch	1	–
	Nomascus leucogenys	1	–
	Symphalangus syndactylus	1	–
Cebidae	Callithrix jacchus	1	1
	Aotus nancymaae	1	1
	Cebus imitator	1	1
	Saimiri boliviensis boliviensis	1	1
	Sapajus apella	1	1
Lemuridae	Lemur catta	1	–
Cheirogaleidae	Microcebus murinus	1	1
Indriidae	Propithecus coquereli	1	1
Lorisidae	Nycticebus coucang	1	1
Galagidae	Otolemur garnettii	1	1

The number of copies of CD300A and CD300C identified in different primate species are indicated and grouped by family. Species with sequences showing clear signs of pseudogenization (e.g., premature stop codons or frameshift mutations) are marked with an asterisk (*). A dash (-) indicates the absence of an identifiable gene copy in the species’ genome.

Our results revealed that the inhibitory receptor CD300A is consistently present across all analyzed species, with a single functional copy. In contrast, CD300C displays a more variable evolutionary pattern. While many species retain a functional gene copy, signs of pseudogenization were detected in *Pan troglodytes*, *Gorilla gorilla*, and *Rhinopithecus bieti.* These pseudogenized CD300C sequences were initially annotated as putatively functional in the NCBI database, with no indication of gene inactivation. However, our alignment-based analyses revealed the presence of premature stop codons in all three. Furthermore, CD300C was completely absent in all analyzed Hylobatidae species and in *Lemur catta*, indicating multiple independent loss events across primate lineages (**Table 1**). This finding is particularly intriguing and raises important questions about the selective forces shaping the evolution of CD300 receptors, as inhibitory receptors like CD300A are often exploited by pathogens to suppress immune responses and evade the host immune system, while activating receptors, such as CD300C, are often thought to arise later in evolution as a countermeasure to these pathogen strategies (7). For instance, human CD300A has been shown to facilitate infection by all four serotypes of Dengue virus, as well as other mosquito-borne viruses including Chikungunya (CHIKV), West Nile virus (WNV), and Yellow Fever virus (18). In parasitic infections, *Leishmania donovani* upregulates CD300A expression in dendritic cells, enhancing the parasite’s ability to establish infection (19, 20). Similarly, in bacterial sepsis models, CD300A-deficient mice exhibit improved bacterial clearance and increased survival compared to wild-type controls (21), further supporting the evidence that CD300A can suppress protective immune responses during infection. Despite this vulnerability to pathogen exploitation, CD300A plays a crucial role in immune regulation. It is essential for maintaining immune homeostasis and controlling inflammation, as shown by the exacerbated inflammatory responses observed in CD300A-deficient mice during antigen-induced arthritis and impaired control of urinary tract infections caused by uropathogenic *Escherichia coli* (22, 23). Moreover, CD300A is required for the resolution of inflammation triggered by monosodium urate (MSU) crystals, through its role in neutrophil apoptosis and efferocytosis. In the absence of CD300A, inflammation becomes prolonged, leading to increased tissue damage (24). These observations underscore the functional duality of CD300A as both a regulatory checkpoint and a potential vulnerability within the immune system. While its inhibitory signaling pathway can be subverted by pathogens to dampen host defenses, CD300A remains indispensable for restraining excessive inflammation and preserving tissue integrity. The remarkable conservation of CD300A across all primate lineages, without any evidence of gene loss or pseudogenization, strongly indicates an evolutionary pressure to maintain this gene and that the benefits of its immunoregulatory role have consistently outweighed the selective disadvantages associated with its exploitation by infectious agents.

### Synteny analysis

**Figure 3:**
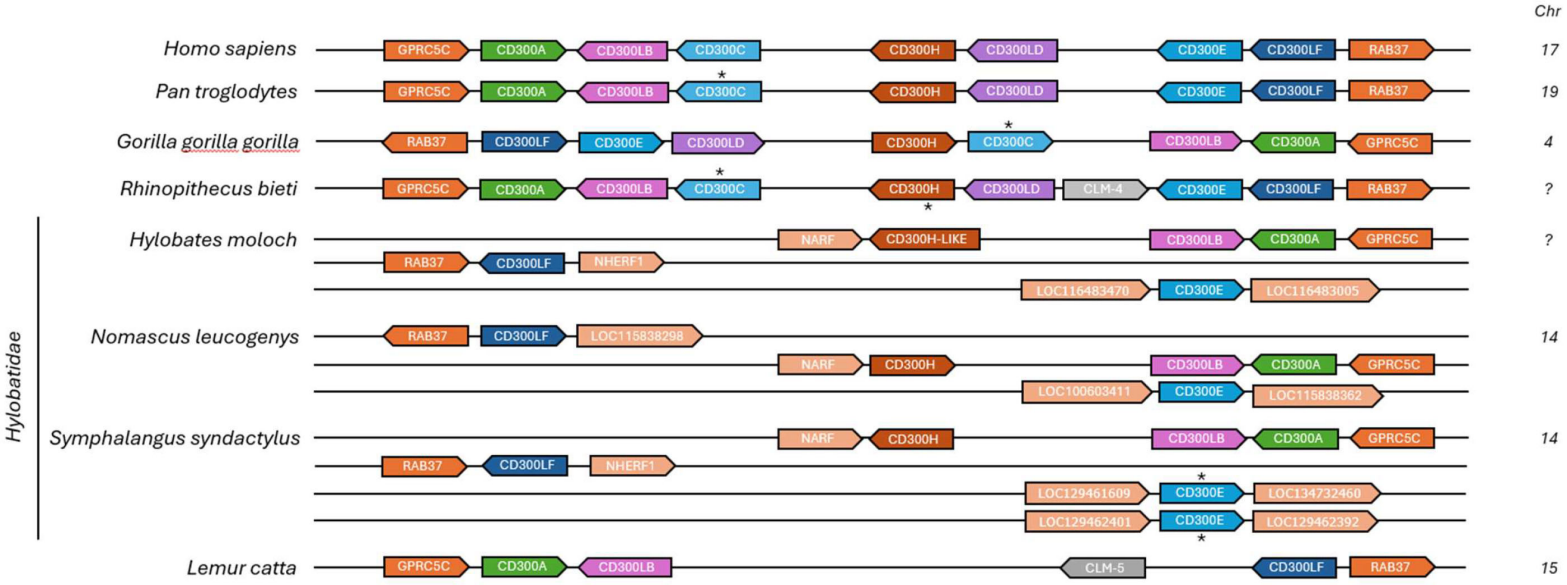
Chromosomal organization of the CD300 gene family in primate species with significant rearrangements and pseudogenization or absence of CD300c compared to human CD300 complex. Each horizontal line and the number in front correspond to the chromosome on which CD300 and flanking genes are located. The asterisk (*) indicates genes that are annotated as pseudogenized in the NCBI database or that we consider pseudogenes based on alignments, in the case of CD300C.

As noted previously (**Table 1**), several primate species exhibit clear signs of CD300C pseudogenization or complete gene loss, contrasting sharply with the strong evolutionary conservation of CD300A. A particularly interesting case is the Hylobatidae family, in which the entire CD300 gene complex exhibits a unique and highly rearranged genomic organization when compared to other primates (**Figure 3**). Multiple CD300 genes, including CD300C, are missing, and others have relocated to different genomic regions, no longer flanked by the typical syntenic markers GPRC5C and RAB37. This pattern strongly suggests that a major chromosomal rearrangement occurred in the common ancestor of Hylobatidae, approximately 9 million years ago (17), leading to the disruption of the original CD300 locus and the subsequent loss of CD300C and other CD300 genes, such as CD300LD.

Genomic rearrangements such as deletions, translocations, and inversions can significantly impact gene families by altering their regulatory landscapes, potentially affecting their expression levels or functional interactions. However, the persistence of this rearrangement across all analyzed Hylobatidae species suggests that it was not a detrimental event but rather one that was either neutral or conferred a selective advantage, indicating that this species did not rely heavily on these receptors or that an alternative immune pathway evolved to have similar functions.

A comparable but independent pattern of CD300C loss is observed in *Lemur catta*, which also lacks CD300C, CD300LD, CD300H, and CD300E. However, unlike Hylobatidae, *L. catta* retains the typical CD300 genomic region, with CD300 genes flanked by GPRC5C and RAB37, indicating that the loss occurred without major chromosomal rearrangements. This reinforces the hypothesis that CD300C loss resulted from an independent event. However, as in Hylobatidae, only activating receptors were lost, while inhibitory members of the CD300 family were preserved. The selective loss of activating receptors such as CD300C in both *L. catta* and Hylobatidae suggests that immune systems in these species may have favored reduced or tightly regulated activation. This may reflect adaptation to a lower pathogen burden, a more stable ecological niche, or the need to prevent immunopathology through hyperactivation. It is also possible that the relocation of some retained CD300 genes in Hylobatidae could allow for new regulatory interactions, placing them under the influence of different promoters, enhancers, or chromatin environments. If these changes resulted in a more efficient immune strategy, conferring greater resistance to pathogens or a reduced risk of immune-mediated tissue damage, they would have been positively selected over evolutionary time.

The evolution of the CD300 gene family in primates follows the birth-and-death molecular evolution. This type of dynamic gene evolution is not unique to the CD300 family. Numerous immune gene families, such as the major histocompatibility complex (MHC) (15, 25), have undergone lineage-specific duplications, losses, and pseudogenization in primates and other mammals. Similar patterns have also been reported for gene families in primates concerned with innate immunity, such as killer cell immunoglobulin-like receptor (KIR) (15, 26), guanylate binding proteins (GBP) (27), tripartite motif (TRIM) (28), and interferon-inducible transmembrane proteins (IFITMs) (29). These families are well-established examples of multigene families evolving under the birth-and-death model. Such an evolutionary pattern is common among immune-related multigene families because it reflects the intense selective pressures exerted by the pathogen-host arms race. In this dynamic conflict, pathogens rapidly mutate to evade immune detection, while hosts counteract through gene diversification via duplications, positive selection, and the elimination or pseudogenization of obsolete variants.

### Analyses of ITIMs regions in CD300A

Previous studies have demonstrated that all four ITIMs contribute to CD300A’s inhibitory function. However, the third ITIM (Y267) is the most important one. Its mutation leads to the loss of most of its ability to inhibit immune responses. Moreover, when the third ITIM is intact, the functional impact of mutations in two of the other ITIMs is limited and doesn’t significantly affect the inhibition of CD300A (11). To evaluate the evolutionary conservation of CD300A ITIMs (**Figure 2A**), sequence alignments were performed across all analyzed primate species (**Figure 2B**). The first three ITIMs follow the canonical consensus motif for classical ITIMs (I/V/L/SxYxxL/V). In the first ITIM (residues 229-234), tyrosine (Y) is conserved across all species, although the initiating residue (I/V/L/S) is replaced by glutamine (Q) and glutamic acid (E) in some species. The second ITIM is the most conserved one, being completely in agreement with the classical ITIM consensus sequence in all the species.

In the third ITIM, three species exhibit a substitution of the initiating residue (I/V/L/S) with proline (P). However, the tyrosine is conserved, and the remaining sequence aligns with the classical ITIM consensus sequence.

The fourth ITIM follows the non-classical consensus motif (I/V/L/S/TxYxxL/V/I). However, this ITIM also shows variability, especially in Lemuriformes and Lorisiformes (*Microcebus murinus*, *Propithecus coquereli*, *Nycticebus coucang*, *Lemur catta*, and *Otolemur garnettii*), where the initial residues (I/V/L/S/Tx) are missing, although the tyrosine itself is preserved. Whether these variations impair inhibitory signaling remains unclear and warrants further functional validation.

### Analyses of ITAMs in CD300C

**Figure 4:**
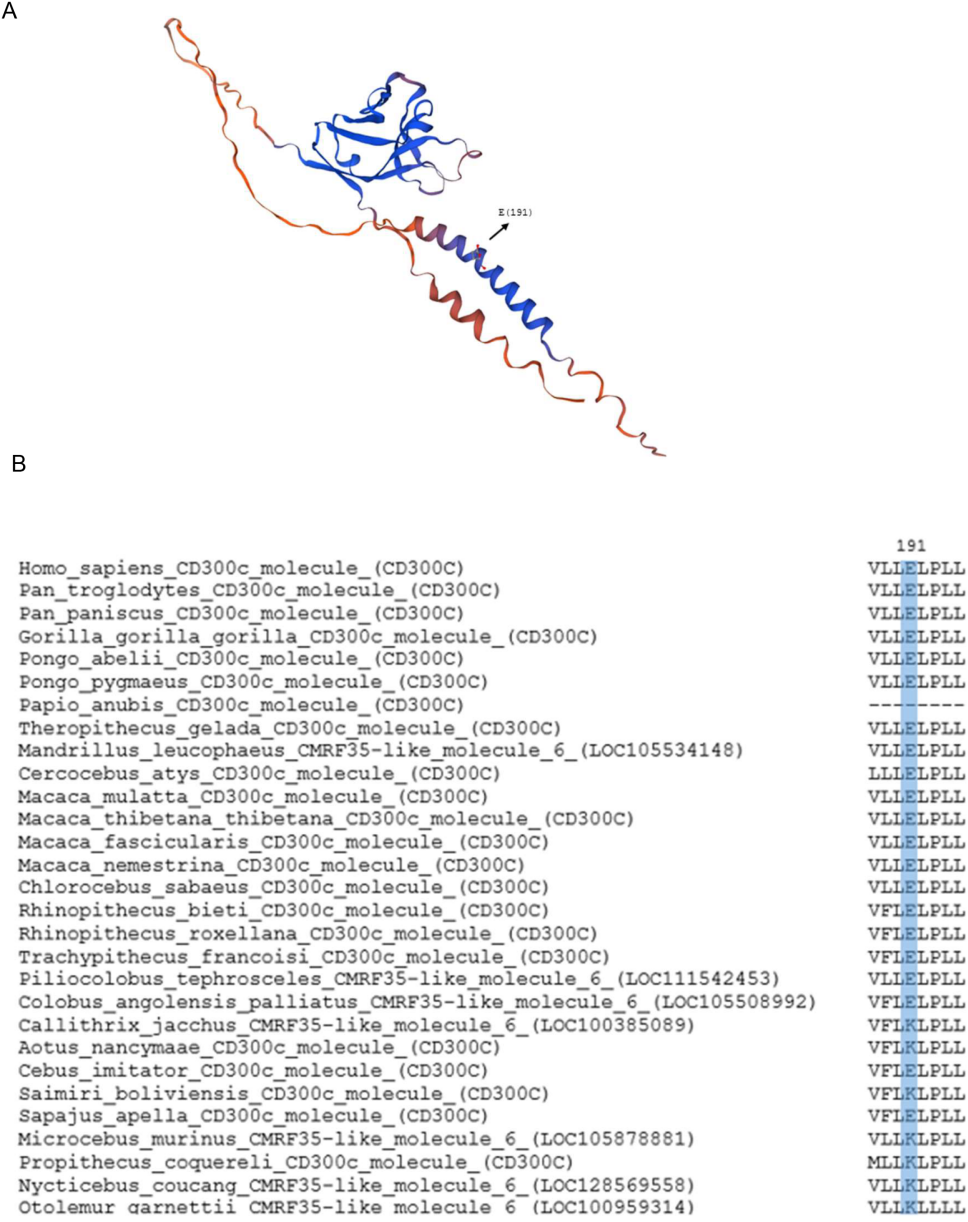
Structure model of human CD300c and conservation of CD300c charged residue responsible for the activating signal. **(A)** Structural model of the human CD300c. The black arrow indicates the position of the charged glutamic acid (E191) in the transmembrane domain responsible for the activating signal of the CD300c molecule. **(B)** Multiple sequence alignment of a segment of the CD300c transmembrane region across various primate species, highlighting the homologous position of the charged glutamic acid residue.

Since CD300C possesses a short cytoplasmic tail lacking ITAMs, its activating signal relies on a charged glutamic acid residue (E191) within the transmembrane domain, which mediates interaction with ITAM-bearing adaptor proteins (**Figure 4A**). Sequence alignments across primate species revealed that the glutamic acid (E191) was replaced by lysine (K) in seven species: three from the Cebidae family (*Callithrix jacchus, Aotus nancymaae, and Saimiri boliviensis boliviensis*) and four others (*Microcebus murinus, Propithecus coquereli, Nycticebus coucang, Otolemur garnettii*) (**Figure 4B**). This substitution may disrupt interaction with adaptor proteins, leading to non-functional or impaired signaling capacity. In principle, lysine (positively charged) could mediate interactions with alternative adaptor proteins that carry complementary negative charge. However, the replacement of the negatively charged glutamic acid with a positively charged lysine may interfere with the electrostatic interactions with the adaptor protein. Activating receptors, such as CD300C, need to associate with specialized signal-transducing transmembrane polypeptides due to the absence of intrinsic signal motifs. This association occurs within the transmembrane domain and depends on the formation of non-covalent interactions between oppositely charged amino acid residues in the receptor and the adaptor molecule (10). Studies have shown that mutating this glutamic acid to a valine (E191V) abolishes signaling through FcRγ, even though the receptor still physically interacts with the adaptor. This suggests that the negative charge of glutamic acid plays a critical role in signal transduction. The study does not directly clarify whether the signaling failure of the E191V mutant is solely due to the loss of negative charge or also due to the introduction of the hydrophobic valine residue. However, the available evidence suggests that the key issue is the loss of the negative charge, as the mutation impairs function despite the structural association with FcRγ being preserved. Therefore, it is plausible to theorize that a substitution by lysine, which introduces a positive charge, may affect the activating signal. However, to determine whether this substitution truly affects receptor function, dedicated functional investigations will be necessary (10). Additionally, the CD300C sequence retrieved from *Papio anubis* was found to be significantly shorter than typical CD300 sequences, lacking both the transmembrane and cytoplasmic domains. This structural deficiency strongly suggests that CD300C in *P. anubis* is non-functional. However, it was not classified as a pseudogene as it did not meet our predefined criteria for pseudogenization, which included the presence of premature stop codons or frameshift mutations. Together, these patterns reinforce the conclusion that CD300C has undergone multiple independent losses or functional impairments across the primate lineage, through both complete gene deletion (as in Hylobatidae and *Lemur catta*) and potential functional loss caused by mutations. This supports a birth-and-death model of evolution, where multigene families expand through gene duplication, but some duplicated genes accumulate deleterious mutations, become pseudogenes, or are eventually lost.

**Figure 5:**
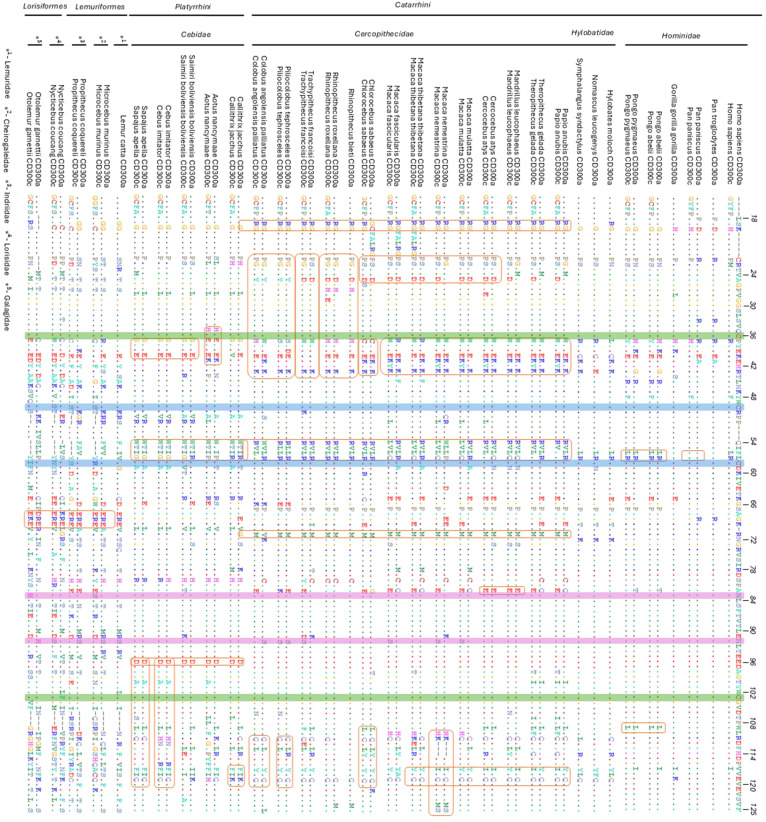
Multiple sequence alignments of the most conserved region of CD300a and CD300c extracellular domains across all primate analyzed species. The sequences are aligned relative to human CD300a to assess conservation and gene conversion events between CD300a and CD300c across primate species and families. The dots (.) indicate residues that are identical to the human CD300a sequence, and the dashes (-) represent gaps in the sequence. Orange boxes highlight regions with potential gene conversion motifs, where sequence similarity between CD300A and CD300c suggests a history of concerted evolution within certain species or primate families. Blue and green boxes highlight disulfide bonds, while the pink boxes highlight N-glycosylation sites. In species where CD300C has been pseudogenized, its sequence has been removed from the alignment.

A comparative analysis of the highly homologous portion of the extracellular domain sequences of CD300A and CD300C (around 112 amino acids) revealed strong evidence of gene conversion across several primate lineages (**Figure 5**). In many species, including humans, both receptors exhibit high sequence similarity in this region, displaying a pattern of concerted evolution, in which sequences from related genes are homogenized through gene conversion, likely preserving the ligand-binding site.

This phenomenon is reflected in the phylogenetic tree (**Supplementary Image 1**), where CD300A and CD300C sequences tend to cluster by species or family rather than by gene type, as typically would be expected. For example, genes from *Piliocolobus tephrosceles* cluster together regardless of receptor type, suggesting that intra-lineage similarity between both sequences in this region exceeds the inter-lineage similarity among orthologs. This suggests that CD300A and CD300C extracellular domains sequence homogenization has occurred preferentially within lineages, rather than maintaining strict divergence between gene copies. Furthermore, the extent of gene conversion between CD300A and CD300C appears to vary across different evolutionary lineages.

This pattern of gene conversion observed in the extracellular domain between CD300A and CD300C is further supported by the presence of unique shared motifs within certain primate families and species (**Figure 5**). For instance, the motif *30*WYEEKHK*36* is exclusive to some Cercophitidae species and is consistently found in both CD300A and CD300C sequences. Similarly, a conserved aspartic acid *93*D residue is present in both genes across all representatives of the Cebidae family, reinforcing the idea of concerted evolution within lineages. This phenomenon also occurs at the species level, for example, in *Aotus nancymaae*, both receptors sequences share the *28*HCEYEEK*34* motif, while in *Macaca nemestrina*, the *107*HK—DPIVQVQVEMS*122* motif is present in both genes, including a three-amino-acid deletion that appears in both sequences. These examples, among many others (**Figure 5**), highlight how gene conversion events maintain sequence similarity between CD300A and CD300C at different taxonomic levels, reinforcing conservation of the ligand-binding region while allowing divergence in the cytoplasmic tail, maintaining their paired receptor dynamic. However, in species where CD300C has been pseudogenized, this pattern of concerted evolution is disrupted. This disruption across multiple independent lineages suggests that the loss of CD300C is not a shared ancestral trait, but rather a repeated outcome of convergent evolution, where similar selective pressures may have driven the parallel inactivation or elimination of this activating receptor.

### Analyses of variation in glycosylation and disulfide bonds sites

The functional constraints and structural features that characterize the CD300A and CD300C genes, essential for receptor stability, are mostly preserved. Cysteine residues (C36↔C103 for CD300A or C43↔C110 for CD300C) involved in disulfide bond formation, which are highlighted in green boxes in **Figure 5**, are highly conserved across most primate species. One notable exception is *Nycticebus coucang* CD300A, where a phenylalanine replaces the cysteine (C→F). The second disulfide bond, corresponding to C57↔C65 in CD300C (blue boxes in **Figure 5**), is also broadly conserved in almost all primates, but is lost in Lemuriformes and Lorisiformes, suggesting lineage-specific alterations that may impact protein stability or ligand-binding efficiency.

Conversely, N-glycosylation sites, highlighted in pink in **Figure 5**, display more variability. Although several glycosylation motifs are retained across families, others are altered in certain lineages and replaced by other amino acids. The most frequent substitution observed is asparagine to serine (N→S), occurring in either CD300A, CD300C, or both. For instance, *Piliocolobus tephrosceles* exhibits shifts in glycosylation sites in both receptors, which may result from gene conversion or adaptive pressures that alter glycosylation patterns. These modifications are particularly evident in some *Cercopithecidae*, *Lemuriformes*, and *Lorisiformes* species, and could influence receptor surface structure, stability, or ligand-binding dynamics.

## Conclusion

Altogether, our findings reveal contrasting evolutionary trajectories for the paired receptors CD300A and CD300C across primates. CD300A is consistently preserved across species, probably reflecting its indispensable role in immune regulation and inflammation control. In contrast, CD300C displays extensive variability, with recurrent pseudogenization, sequence degradation, or complete loss in multiple independent lineages. This pattern suggests that CD300C is subject to lineage-specific selective pressures where its function may be dispensable or replaceable. The occurrence of gene conversion signals between CD300A and CD300C in species retaining both genes further illustrates the dynamic interplay between these paralogs. Such events likely preserve ligand-binding capacity despite functional divergence in downstream signaling.

Despite the comprehensive nature of this analysis, some limitations remain. The selective pressures underlying the repeated loss or inactivation of CD300C across multiple lineages remain unclear. Factors such as pathogen diversity, immune response, and ecological niche may all influence receptor retention and require further investigation. Additionally, differences in genome assembly quality and gene annotation inconsistencies across species may confuse interpretations of gene structure and function. Given the clustered organization and high sequence similarity among CD300 genes, short-read sequencing and fragmented assemblies can lead to missing regions, misassembled loci, or misannotated pseudogenes. This can affect analyses such as gene copy number variation, structural variations (like insertions and deletions), and phylogenetic inference. Therefore, some of the observed gene absences or pseudogenization events, especially in genomes of lower quality, should be interpreted carefully, since they may result from incomplete assemblies or annotation errors and may require future validation with higher-quality genome assemblies or transcriptomic data. In future research, it would be interesting to include functional assays to test the signaling activity of divergent or truncated receptor variants, expression studies to confirm transcriptional activity, and comparative immunological studies across species to explore whether alternative activating pathways have emerged in the absence of CD300C.

## Data Availability

The original contributions presented in the study are included in the article/**Supplementary Material**. Further inquiries can be directed to the corresponding author.
