# Supplementary material for: Evolutionary analysis of CD300A and CD300C paired receptors in primates
Source: Front Immunol. 2025 Sep 5;16:1633323. doi: 10.3389/fimmu.2025.1633323 (PMC12447643; doi:10.3389/fimmu.2025.1633323)
Supplement: Supplementary file 1 [file Table1.docx]

**Supplementary data**

**
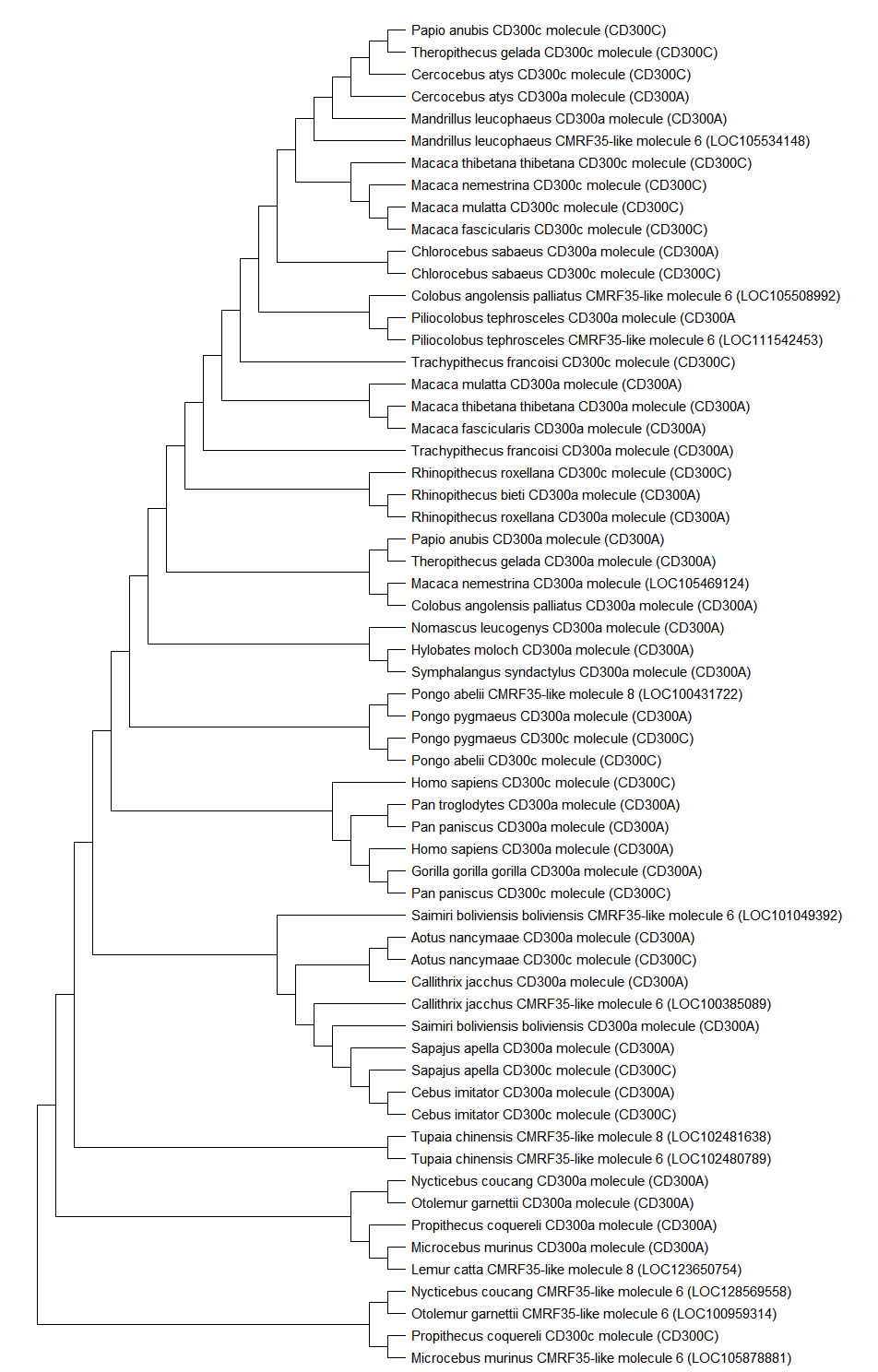
**

**Supplementary figure 1 – Maximum-likelihood (ML) phylogenetic tree of the conserved region of the extracellular domain between CD300a and CD300c sequences.** The phylogenetic tree was constructed using the JTT+G nucleotide substitution model with five gamma-distributed rate categories.
